# Plant growth promotion induced by phosphate solubilizing endophytic *Pseudomonas* isolates

**DOI:** 10.3389/fmicb.2015.00745

**Published:** 2015-07-22

**Authors:** Nicholas Oteino, Richard D. Lally, Samuel Kiwanuka, Andrew Lloyd, David Ryan, Kieran J. Germaine, David N. Dowling

**Affiliations:** Department of Science and Health, EnviroCore. The Dargan Research Centre, Institute of Technology CarlowCarlow, Ireland

**Keywords:** plant growth promotion, *Pseudomonas fluorescens*, endophytes, PQQ, *Pisum sativum* L

## Abstract

The use of plant growth promoting bacterial inoculants as live microbial biofertilizers provides a promising alternative to chemical fertilizers and pesticides. Inorganic phosphate solubilization is one of the major mechanisms of plant growth promotion by plant associated bacteria. This involves bacteria releasing organic acids into the soil which solubilize the phosphate complexes converting them into ortho-phosphate which is available for plant up-take and utilization. The study presented here describes the ability of endophytic bacteria to produce gluconic acid (GA), solubilize insoluble phosphate, and stimulate the growth of *Pisum sativum* L. plants. This study also describes the genetic systems within three of these endophyte strains thought to be responsible for their effective phosphate solubilizing abilities. The results showed that many of the endophytic strains produced GA (14–169 mM) and have moderate to high phosphate solubilization capacities (~400–1300 mg L^−1^). When inoculated into *P. sativum* L. plants grown in soil under soluble phosphate limiting conditions, the endophytes that produced medium-high levels of GA displayed beneficial plant growth promotion effects.

## Introduction

Phosphorus is the second most important nutrient for plants, after nitrogen. It exists in soil as mineral salts or incorporated into organic compounds. Despite these phosphorus compounds being abundant in agricultural soils, the majority of them occurs in an insoluble form (Miller et al., 2010). Plants require approximately 30 μmol l-1 of phosphorus for maximum productivity, but only about 1 μmol l-1 is available in many soils. Therefore, the unavailability of phosphorus in many soils has been recognized as a major growth limiting factor in agricultural and horticultural systems (Daniels et al., 2009). This necessitates the application of soluble forms of phosphorus in the form of phosphate fertilizers, which in itself has constraints in that it too is rapidly immobilized (fixed) to insoluble forms upon its application in the soil due to its reaction with aluminum and iron minerals. The efficiency of applied phosphorus rarely exceeds 30% due to fixation in soil (Sharma et al., 2013). It is also lost as a result of run-off and leaching, leaving as little as 10–20% available for plant utilization (Sashidhar and Podile, 2009). Phosphate fertilizers are dependent on phosphorus derived from phosphate rock, which is a non-renewable resource and current global reserves may be depleted in 50–100 years (Cordell et al., 2009). Therefore, exploring alternative forms of agriculture, where nutrient conservation is key, is of vital importance.

Several reports have indicated that different bacterial species, particularly rhizosphere colonizing bacteria, have the ability to liberate organic phosphates or to solubilize insoluble inorganic phosphate compounds such as tricalcium phosphate, dicalcium phosphate, hydroxyapatite, and rock phosphate. These bacteria make available the soluble phosphates to the plants, and in return gain root borne carbon compounds, mainly sugars and organic acids, necessary for bacterial growth (Khan et al., 2010). Current research suggests that the inoculation of crops with Phosphate Solubilizing Microbes (PSM) has the potential to reduce application rates of phosphate fertilizer by 50% without significantly reducing crop yield (Jilani et al., 2007; Yazdani et al., 2009). Phosphate Solubilizing Bacteria (PSB) may also be useful in the phyto-remediation of heavy metal impacted soil (Ahemad, 2015; Monica and Harshada, 2015) or for bioleaching of rare Earth elements for mined ores (Shin et al., 2015).

The liberation of organic phosphates by bacteria is mediated through the production of enzymes such as phytases, C-P lyases, and phosphonatases. The principal mechanism for mineral phosphate solubilization is the production of organic acids and acid phosphatases (Illmer et al., 1995). In organic acid production mechanisms, gluconic acid (GA) seems to be the most frequent agent of inorganic phosphate solubilization and to a lesser extend α-ketogluconic acid (Puente et al., 2004; Rodriguez et al., 2006). In many reports, the acids are produced in the periplasm of Gram- negative bacteria by a direct oxidation pathway of glucose (DOPG; non-phosphorylating oxidation) (Anthony, 2004). In the DOPG, the enzyme glucose dehydrogenase (GCD/GDH) and gluconate dehydrogenase (GAD) orient to the outer face of the cytoplasmic membrane and are able to oxidize the substrate in the periplasmic space (Chhabra et al., 2013). As a result, the organic acids diffuse freely outside the cells releasing high quantities of soluble phosphate from mineral phosphates, by supplying both protons and metal complexing organic acid anions (Rodríguez and Fraga, 1999).

GA biosynthesis is commonly carried out by the enzyme glucose dehydrogenase (GCD) in the presence of the cofactor, pyrroloquinoline quinone (PQQ) (Sharma et al., 2013). PQQ is a small, redox active molecule that serves as a cofactor for several bacterial dehydrogenases. The production of the PQQ molecule is encoded in the *pqq* operon which consists of six core genes *pqqA,B,C,D,E*, and *F*, of which PqqA, PqqC, PqqD, and PqqE are essential. PqqA is a small 22–24 amino acid peptide which acts as a substrate for PqqE. PqqC is a cofactor less, oxygen-activating enzyme catalyzing the final step in PQQ biosynthesis. The function of PqqD is not fully understood, but it has recently been shown to interact physically with PqqE. PqqE is a functional radical S-Adenosyl-L-methionine (SAM) enzyme capable of catalytic reductive cleavage of SAM to methionine and 5'-deoxyadenosine. PqqB is suspected to be a member of the metallo-β-lactamases family of proteins and PqqF is a peptidase, but these two proteins are not essential for the production of PQQ (Shen et al., 2012).

This paper describes the phosphate solubilization ability, GA production, plant growth promotion abilities (under soluble phosphate limiting conditions) and the genes thought to be involved in mineral phosphate solubilization in three endophytic isolates from the bioenergy crop *Miscanthus giganteus*. These three *Pseudomonas fluorescens* isolates have been shown to have excellent plant colonization abilities, particularly of the rhizosphere, and potent plant growth promotion capabilities (Oteino et al., 2013). They therefore represent good candidate microbes to be used as commercial biofertilizer strains.

## Materials and methods

The bacterial cultures used in this study are detailed in Table 1.

**Table 1:** **Bacterial cultures and genomes used in this study**.

**Strain No**.	**Original Source**	**ID**	**References/Accession no**.
L111	*Miscanthus giganteus* (leaf endophyte), Ireland.	*Pseudomonas fluorescens*	Keogh, 2009
L228	*Miscanthus giganteus* (leaf endophyte), Ireland.	*Pseudomonas fluorescens*	Keogh, 2009
L321	*Miscanthus giganteus* (leaf endophyte), Ireland.	*Pseudomonas fluorescens*	Keogh, 2009
L132	*Miscanthus giganteus* (leaf endophyte), Ireland.	*Pseudomonas* sp.	Keogh, 2009
S20	*Brassica napus* (stem endophyte), Ireland.	*Bacillus* sp.	Odhiambo, 2011
F113	*Beta vulgaris* (rhizosphere isolate), Ireland.	*Pseudomonas fluorescens*	Fenton et al., 1992 GCF_000237065.1
JM109		*E. coli*	Promega Ltd
SBW25	*Beta vulgaris* (phylosphere isolate), U.K.	*Pseudomonas fluorescens*	Silby et al., 2009 NC_012660.1
SS101	*Triticum* sp. (rhizosphere isolate), The Netherlands.	*Pseudomonas fluorescens*	Loper et al., 2012 NZ_CM001513.1
A506	*Pyrus* sp. (phylosphere isolate), U.S.A.	*Pseudomonas fluorescens*	Loper et al., 2012 NC_017911.1
Pf-5	Soil isolate U.S.A.	*Pseudomonas protegens*	Paulsen et al., 2005 NC_004129.6
NCIMB 11764	Soil isolate U.K.	*Pseudomonas fluorescens*	Vilo et al., 2012 GCF_000293885.1
UW4	Common Reed (rhizosphere isolate), U.S.A.	*Pseudomonas fluorescens*	Duan et al., 2013 NC_019670.1
W619	*Populus trichocarpa* × *P. deltoides* (endophyte), U.S.A.	*Pseudomonas putida*	Taghavi et al., 2009 NC_010501.1

### Phosphate solubilization bioassay and determination of gluconic acid in the culture medium

The isolates were individually grown in LB broth medium overnight and the OD 600 nm adjusted to 1.0. The cells were washed twice in 0.85% sterile ringers before inoculating in National Botanical Research Institute's Phosphate (NBRIP) (Nautiyal, 1999) growth medium containing insoluble tricalcium phosphate (Ca_3_(PO_4_)_2_). The pH of the NBRIP media was adjusted to 6.75 ± 0.25 before autoclaving. The strains were inoculated in 20 ml vials containing NBRIP media and incubated at 30°C in a shaker incubator (150 rpm) for 5 days. Autoclaved, un-inoculated NBRIP medium and media inoculated with *E. coli* JM109 served as negative controls. Due to the presence of suspended particles of insoluble Ca_3_(PO_4_)_2_ in the supernatant, the broths were centrifuged at 13,000 rpm for 10 min to obtain a clear supernatant. Triplicate aliquots of the supernatant (100 μl) were transferred into clean, dry, acid washed test tubes. Soluble phosphate was determined using the Fiske and Subbarow method (Fiske and Subbarow, 1925). Briefly, a 4.2 ml volume of double distilled water was added to each tube, in addition to 500 μl of ammonium molybdate (2.5%) solution and 200 μl of α-amino-naphthol solution prepared with 1-amino-2-naphthol-4 sulphonic acid. The tubes were vortexed and incubated at room temperature for 30 min. Thereafter, the solution was read at 660 nm using a spectrophotometer and the level of phosphate was estimated by extrapolating against the prepared phosphate standard curve. A subsample of this supernatant was used to determine the final pH and GA concentration of each sample. The determination of GA was carried out by High Performance Liquid Chromatography (HPLC) Shimadzu Prominence using a C_18_ column (250 × 4.6 mm) set at the following parameters; solvent 20% methanol and 80% deionized sterilized H_2_O; flow rate 0.8 ml/min; temperature 40°C; UV detector 210 nm and injection volume 50 μl. The supernatants were filtrated through a 0.22 μm filter prior to analysis. The triplicate samples were then analyzed on a single run on the HPLC. Autoclaved un-inoculated medium and *E. coli* inoculated media served as control. To determine which organic acids were produced by the strains authentic standards of acetic, gluconic, α-ketogluconic propionic, lactic, citric, malic, succinic, and pyruvic acids were used in the HPLC assay. GA was quantified by reference to the retention time (3.25 min) and peak area chromatograph obtained for authentic standard for GA (Sigma-Aldrich, Dublin) with a concentration range between 0 and 120 mM.

### Mobilization of phosphorus to pea plants by endophytic bacterial strains

An experiment to study the phosphorus mobilization to plants by selected endophytic bacteria (L321, L132, and S10; representing high, medium, and low P solubilizing ability, respectively) was conducted in pots under greenhouse conditions using *P. sativum* L. var Early Onward. Horticultural sand was used as the growth substrate in these experiments. The sand was washed with distilled water and rinsed several times to remove any trace of soluble phosphate and then left to dry in the open air at room temperature for 7 days. The dried sand was then thoroughly mixed with insoluble tricalcium phosphate (Ca_3_(PO_4_)_2_) in a 200:1 (wt/wt) ratio before use. 250 g of this sand was placed into plastic plant growth pots.

*P. sativum* L. seeds were inoculated with individual bacterial strains by coating them in a bacteria-calcium alginate mix as described by Power et al. (2011). Briefly, cells from 10 ml overnight cultures were harvested by centrifugation at 10,000 rpm and resuspended in 20 ml sterile 4% sodium alginate gel. *P. sativum* L. seeds were coated in this gel and then dropped into 2% calcium chloride solution for 10 min. The coated seeds were harvested and washed twice in sterile water. Four seeds of *P. sativum* L. encapsulated with appropriate bacterial inoculum were sown in each pot. The seedlings were later thinned to two plants per pot, 2 weeks after sowing. The experimental treatments consisted of triplicate pots containing; (a) inoculated seeds; (b) un-inoculated seeds as a negative control and (c) un-inoculated seeds sown in sand [with no (Ca_3_(PO_4_)_2_ amendment] which were watered regularly with plant nutrient solution (International Organisation for Standardisation (ISO 8692), 1997) containing soluble phosphate (0.05 M KH_2_PO_4_). All plants were cultivated in the greenhouse (16 h day/8 h night, mean air temperature 22 ± 3°C). The plants were watered twice per week with 15 ml plant nutrient solution in the presence or absence of soluble phosphate where applicable. Harvesting was carried out after 60 days of growth. Roots were washed under tap water, and root and shoot fresh and dry weights were determined.

### Bioinformatic analysis of phosphate solubilization systems in selected endophytic strains

The *pqq* operon and *gcd* genes in *Pseudomonas fluorescens* L321, L111, and L228 were identified in preliminary genome sequencing data (Dowling, unpublished data). The sequence data of these *pqq* and *gcd* genes were submitted to Genbank and can be found under accession numbers KP981419, KP981420, KP981421, KR002856, KR002857, and KR002858.

Phylogenetic analysis were performed using MEGA6 software (Tamura et al., 2013). Amino acid sequences obtained from the NCBI database were concatenated in a FASTA formatted file and uploaded to MEGA6 software. Protein alignments were performed using the ClustalW (Thompson et al., 1994) function, using an adjusted multiple alignment gap opening penalty of three and a gap extension penalty of 1.8 (Hall, 2013). Evolutionary phylogenetic relationship for each strain was determined by aligning the predicted Pqq protein sequences concatenated in the order PqqF, PqqA, PqqB, PqqC, PqqD, and PqqE. Phylogeny was constructed using the Neighbor-joining method (Saitou and Nei, 1987), with 1000 bootstrap replications using partial deletion of gaps/missing data with a site coverage cut off of 95%. Details of *Pseudomonas* strains used in the alignment are described in Table 1. MEGA6 software (McWilliam et al., 2013) was used to generate a graphic of the sequence alignment.

### Statistical analysis

Experimental data were analyzed statistically using ANOVA. Significance of the effect of treatment was determined by the magnitude of the *F*-value (*P* < 0.05). When a significant *F*-test was obtained for the treatments, separation of means was accomplished by Fisher's protected LSD. Statistical analysis of the results were performed using general linear model (GLM) in R statistics version 3.0.0 (2013) and the means were separated by Newman-Kuel test. The significance level was set at ≤0.05.

## Results

### Inorganic phosphate solubilization and analysis of gluconic acid production

After incubation of the endophytic strains for 5 days at 30°C, the strains showed great variation in phosphate-solubilization capacity (Table 2). Solubilized phosphate estimated in the NBRIP supernatant varied from 85 mg to 1312 mg L^−1^ with the highest solubilization recorded in L228 and L132. The lowest level of solubilized phosphate was recorded in S20 and L111. In the negative control, *E. coli* JM109, solubilized phosphate was not detected in the supernatant. The culture supernatants were analyzed by HPLC in order to determine if organic acids were produced by the strains. All strains showed production of GA with a concentration ranging from 2840 to 33240 ± 230 mg L^−1^ (14–169 mM). Although other minor peaks did appear in the HPLC chromatographs, none of the retention times of these peaks corresponded to those of the other organic acids tested and in all cases represented <1% of the peak area of the GA peak. Strains F113, L132, and L321 produced similar high levels of GA while strain L111 and S20 produced relatively low levels. With the exception of strain L228, the drop in pH of the bacterial supernatants corresponded to the levels of GA detected. Generally, culture medium with low pH recorded a high available soluble phosphate and GA production.

**Table 2:** **Phosphate solubilization, gluconic acid production, and pH values of endophytic strains in NBRIP broths (means ± standard deviations, ***n*** = 3) ND, Not determined**.

	**Mean soluble P (mg L/^−1^)**	**Gluconic acid (mg L/^−1^)**	**pH**
L111	438±1.8	4940±180	4.96
L132	1024±1.7	31490±3710	4.43
L228	1312±4.1	22200±320	4.06
L321	788±0.4	33240±2340	4.44
S20	85±0.3	2840±200	5.28
F113	649±0.2	33330±300	4.35
JM109	ND	0.00±300	6.5

### Mobilization of phosphorus to pea plants by endophytic bacterial strains

**Figure 1:**
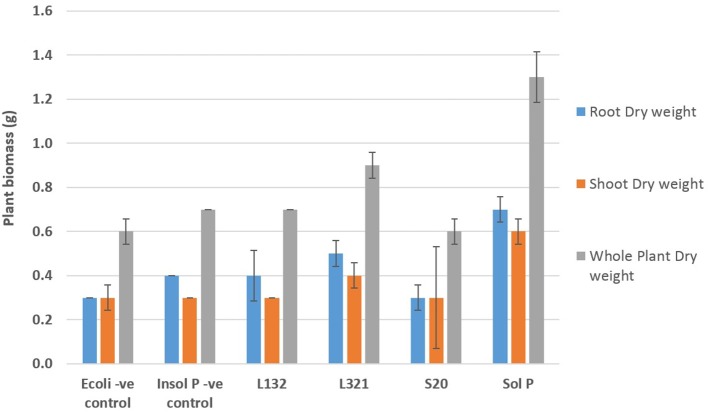
**Biomass (dry weight) of ***P. sativum*** L. plants inoculated with bacterial endophytic strains capable of solubilizing inorganic phosphate (Ca_3_(PO_4_)_2_) after 60 days of cultivation under greenhouse conditions**. Insol P, Insoluble phosphate; Sol P, Soluble phosphate. *E. coli* (–ve cont), soil inoculated with *E. coli* and amended with insoluble phosphate. Bars represent the mean of 10 replicate pots (*n* = 10), error bars represent the standard error of the mean.

Varied results were observed for the growth parameters in *P. sativum* L. plants among the endophyte inoculated treatments and controls (Figure 1). As expected un-inoculated plants treated with the soluble phosphate (positive control) produced the highest quantity of biomass (total weight, root weight, and shoot weight). The growth in these positive controls was significantly greater than that seen in all of the other treatments. Un-inoculated plants grown in sand containing insoluble phosphate (the negative control) produced the least biomass. Among the plants treated with the endophytes, plants inoculated with strain L321 significantly outperformed all three of the other bacterial treatments for both fresh weight and dry weight measurements. Enhanced plant growth was also observed in plants inoculated with strain L132, and to a lesser extent plants inoculated with S10 and *E. coli* JM109. A significant difference was recorded in the root and shoot dry weight in plants treated with L321 (*b* = 0.2, *t* = 2.22, *p* = 0.03) compared with the rest of the inoculated treatments. The highest whole plant dry weight, 0.88 ± 0.13 g, was also recorded in plants treated with L321 and represented an increase of 1.44 fold over the negative control.

### Bioinformatic analysis of gluconic acid production systems in selected endophytic strains

**Figure 2:**
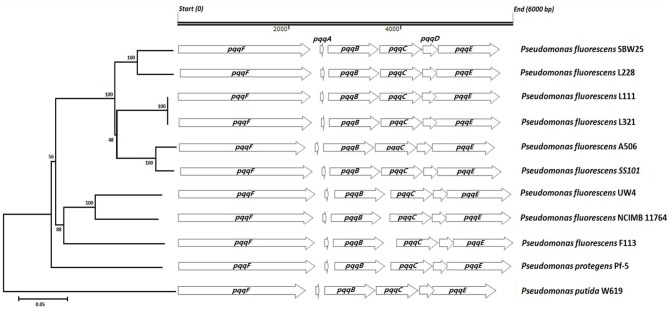
**Neighbor-joining phylogenetic tree predicting the relationships among the ***pqq*** operons in strains L228, L321, and L111 with those of published ***Pseudomonas*** genomes**. The tree was constructed using the concatenated protein products in the order PqqF, PqqA, PqqB, PqqC, PqqD, and PqqE (bootstrap values presented at the nodes).

Nucleotide sequence analysis of the *pqq* operon in *Pseudomonas fluorescens* strains L321, L111, and L228 showed that all three strains had the operon with gene order of *pqqFABCDE* (Figure 2). In strains L111, L321, L228 and many of the other *Pseudomonas* genomes examined there was a predicted overlap of 1 codon between the *pqqC* and *pqqD* gene. The sizes of the proteins in all six genes of the operon were consistent with those of other *Pseudomonas pqq* genes (Table 3).

**Table 3:** **Genes implicated in the phosphate solubilization abilities of ***Pseudomonas fluorescens*** strains L228, L321, and L111**.

	***Pseudomonas fluorescens* L321**				***Pseudomonas fluorescens* L111**				***Pseudomonas fluorescens* L228**			
**Gene**	**Gene #**	**Genomic Location**	**Len AA**	**Strand**	**Gene #**	**Genomic Location**	**Len AA**	**Strand**	**Gene #**	**Genomic Location**	**Len AA**	**Strand**
*gcd*	1232	1135886	803	+	1287	1196143	803	+	1165	1078762	803	−
*pqqF*	6605	5995918	793	−	6587	5959138	793	−	6052	5455064	782	+
*pqqA*	6605	5993371	24	−	6587	5958978	24	−	6052	5457623	24	+
*pqqB*	6604	5993255	303	−	6586	5957921	303	−	6053	5457764	303	+
*pqqC*	6602	5992302	250	−	6584	5957157	250	−	6055	5458687	250	+
*pqqD*	6601	599155	91	−	6583	5956885	91	−	6056	5459436	91	+
*pqqE*	6600	5991306	393	−	6582	5955762	393	−	6057	5459683	383	+

In nearly all of the genomes examined, there was a predicted overlap of 3–20 codons between the *pqqD-E* genes (L111, L321 showed a 20 codon overlap while L228 has a 10 codon overlap between the *pqqC* and *pqqD* gene). These predicted overlaps were not in the same reading frame. Phylogenetic analysis shows that there was 100% amino acid sequence identity between the PQQ proteins from *Pseudomonas fluorescens* strains L111 and L321, while the *pqq* operon in *Pseudomonas fluorescens* L228 was different and clustered with *Pseudomonas fluorescens* SBW25. Phylogenetic analysis of the glucose dehydrogenase protein (GDH) (supplementary information) also showed that they were more closely related in L321 and L111 than they were with those in strain L228. There was less genetic variation in the core *pqq* genes (*pqqABCDE*) than there was in *pqqF*. PqqF is not essential for the production of PQQ. The phylogenetic tree of the concatenated *pqq* operon proteins shows two main clusters. *Pseudomonas* strains L228, L321, L111, A506 and SS101 and SBW25 formed one cluster while *Pseudomonas* strains F113, Pf-5, NCIMB11764, and UW4 formed another cluster. These two clusters were consistent with the presence or absence of a 139–204 bp intergenic region between the *pqqB* and *pqqC* genes. The phylogenetic tree was constructed based only on the protein coding sequences of the *pqq* regions. However, it still produced a tree that resulted in the clustering of strains that contained this intergenic region. *Pseudomonas putida* W614 formed an out-group both in the concatenated tree and trees created for each individual gene in the *ppq* operon and the *gcd* gene (Supplementary Data) suggested that this strain has a very different evolutionary history. The gene encoding GAD was detected in all three endophytic stains. As α-ketogluconic acid was not detected in the HPLC analysis, it may have little involvement in mineral phosphate solubilizing in these strains.

## Discussion

In the rhizosphere, bacteria secrete organic acids which results in phosphate solubilization from insoluble complexes, making it available for plant uptake (Richardson et al., 2009). One of the most important phosphate solubilization mechanisms in plant associated bacteria is the production of low molecular weight organic acids which results in the acidification of the soil or media (Gyaneshwar et al., 1999; Puente et al., 2004; Khan et al., 2014). These organic acids can chelate the cation bound to phosphate with their hydroxyl and carboxyl groups (Kpomblekou and Tabatabai, 1994). The most efficient mineral phosphate solubilization phenotype in Gram-negative bacteria results from extracellular oxidation of glucose to GA via quinoprotein glucose dehydrogenase (Hilda et al., 2000). In this study, six plant-associated bacteria were tested for their ability to solubilize tricalcium phosphate (Ca_3_(PO_4_)_2_) and their ability to produce GA. Five out of the six strains tested were able to solubilize Ca_3_(PO_4_)_2_ to the level of >400.00 mg L^−1^. All five of these strains were *Pseudomonas* sp. while the sixth strain, which showed poor phosphate solubilization, was a *Bacillus* strain. Typical phosphate solubilization values in PSB range from 10 to 800 mg L^−1^ (Rodríguez and Fraga, 1999; Stephen and Jisha, 2011; Hussain et al., 2013; Surapat et al., 2013). Two strains (L228 and L132) in the current study were found to be very effective PSBs, solubilizing in excess of 1000 mg L^−1^. A relationship between supernatant acidity, the concentration of phosphate available in the supernatant and the concentration of GA produced was observed. In general, the greater the level of GA produced the higher the concentration of phosphate was released into the media. Acidification seemed to be the main strategy utilized by these strains for solubilizing phosphate. HPLC analysis of the supernatants showed that large concentrations of GA were released into the media. Minor quantities of other organic acids may also have been released into the media as there were other smaller unidentified peaks present in the chromatographs. Surapat et al. (2013) found that the PSBs they were studying produced mainly GA although they did detect other organic acids such as lactic, acetic, succinic, propionic, and citric acids. GA production in the endophytic bacteria in the current study ranged from 14 to 169 mM after 5 days. Mardad et al. (2013) found that their PSB strains produced 44–55 mM GA after 7 days. When inoculated into the rhizosphere of *P. sativum* L. plants under soluble phosphate limiting conditions, the strains that were capable of producing medium-high levels of GA, resulted in greater plant growth promotion ability. The results showed that *P. sativum* L. plants treated with endophytic *Pseudomonas* fluorescens strains L321 or L132, had increased plant fresh weights and dry weights. In particular, plants inoculated with strain L321 consistently showed significant positive effects on plant growth parameters over un-inoculated *P. sativum* L. plants. This suggested that this strain can solubilize the insoluble phosphate compound present in the sand medium resulting in plant growth promotion. Plants treated with the *E. coli* JM109 strain (negative control), that did not produce GA, did not show significant plant growth promotion. Increased plant growth and phosphate uptake have been reported in many crop species as a result of PSB inoculants, e.g., *Pseudomonas* sp. in rice (Gusain et al., 2015), *Pseudomonas* in soya bean (Fankem et al., 2015) and *Pseudomonas* sp. in wheat (Babana and Antoun, 2006). Demissie et al. (2013) showed that the inoculation of faba bean (*Vicia faba* L.) with *Pseudomonas* and *Rhizobium* isolates in the presence and absence of phosphate sources significantly (*p* < 0.05) increased plant height compared to the control. Hussain et al. (2013) investigated five promising strains of PSB [PS-01 (*Burkholderia* sp.), PS-12 (*Bacillus* sp.), PS-32 (*Pseudomonas* sp.), PS-41 (*Flavobacterium* sp.), and PS-51 (*Pseudomonas* sp.)] and found that they significantly increased plant height, root length, shoot dry weight, root dry weight, and grain yield up to 16, 11, 42, 29, and 33%, respectively, over the un-inoculated control. Surapat et al. (2013) found that the inoculation of chili plants (*Capsicum frutescens* L.) significantly enhanced plant growth and phosphate uptake when compared to un-inoculated plants. Walpola and Yoon (2013) showed a drop in soil pH and an increase in soluble phosphate after the addition of PSB. They also showed a significant increase in the growth of mung beans [*Vigna radiata* (L.) Wilczek] after inoculation with PSB.

Genetic analysis of the three endophytic strains L228, L321, and L111 showed that they all possessed a full *pqq* operon and both *gcd* and *gad* genes. The *pqq* operon gene order was *pqqFABCDE* in all three endophytic strains. This order is found in about 40% of PQQ producing species. However, in many other *Pseudomonas* species the gene order in the *pqq* operon is *pqqABCDEF*. Also in most other bacterial species the *pqq*F gene is remote from the *pqqA-E* cluster (Shen et al., 2012). Presence of this operon and the associated *gcd* gene provides further evidence that in these three strains production of GA is a major mechanism for solubilizing phosphate. Choi et al. (2008) demonstrated that PQQ itself could be responsible for plant growth promotion. They treated cucumber (*Cucumis sativus* L.) plants with various doses of synthetic PQQ which resulted in plant growth promotion in a dose dependent manner. This plant growth promotion was observed in both hydroponic and soil based systems and suggests that the production of PQQ itself could itself be considered to be a plant growth promoting trait (Misra et al., 2012).

The current study shows that the inoculation of plants with PSB, grown under soluble phosphate limiting conditions, resulted in greater plant growth, than un-inoculated plants. It is proposed that these inocula produced GA in the rhizosphere of the inoculated plants that resulted in the release of soluble phosphate and that this soluble phosphate was subsequently assimilated by the plant. However, these bacteria are also known to express other plant growth promotion traits such as indole-3-acetic acid production and aminocyclopropane-1-carboxylic acid de-aminase activity which may also have contributed to the enhanced growth of the inoculated plants. It is also possible that the production and release of PQQ by the inoculated strains may have enhanced the phosphate solubilizing activity of other indigenous microflora. Ideally, to confirm the involvement of both PQQ and glucose dehydrogenase in phosphate solubilization within the rhizosphere, gene expression analysis of these systems would need to be conducted. Although, this was not part of the current study, *in situ* expression of the PQQ and glucose dehydrogenase genes in phosphate solubilizing *Pseudomonas* strains has recently been shown in the rhizosphere of inoculated maize plants. Rice et al. (2012) created two *lux* fusion biosensor strains, one based on the *gcd* gene and the other on the *pqq*B gene. They were able to detect expression of both genes in the maize rhizosphere and showed that expression was significantly greater in one cultivar than in the other and that this correlated with the colonization abilities of the strains in both cultivars.

The use of plant growth promoting bacteria inoculants as biofertilizer provides a promising alternative/amendment to chemical fertilizers. The availability of soil microorganisms to convert insoluble forms of phosphorus to a soluble form is an important trait in plant growth promoting bacteria for increasing yields. The results described in this publication show that endophytic *Pseudomonas* strains L111, L228, and L321 possess good phosphate solubilization activity and in the case of L321 this trait was expressed under phosphate limiting conditions resulting in enhanced plant growth promotion of *P. sativum* L. plants. These *Pseudomonas* strains, and in particular *Pseudomonas fluorescens* L321, may be ideal live microbial biofertilizer candidates for commercial applications.

### Conflict of interest statement

The authors declare that the research was conducted in the absence of any commercial or financial relationships that could be construed as a potential conflict of interest.
