# Supplementary material for: Plant growth promotion induced by phosphate solubilizing endophytic Pseudomonas isolates
Source: Front Microbiol. 2015 Jul 22;6:745. doi: 10.3389/fmicb.2015.00745 (PMC4510416; doi:10.3389/fmicb.2015.00745)
Supplement: Supplementary file 1 [file DataSheet1.DOCX]

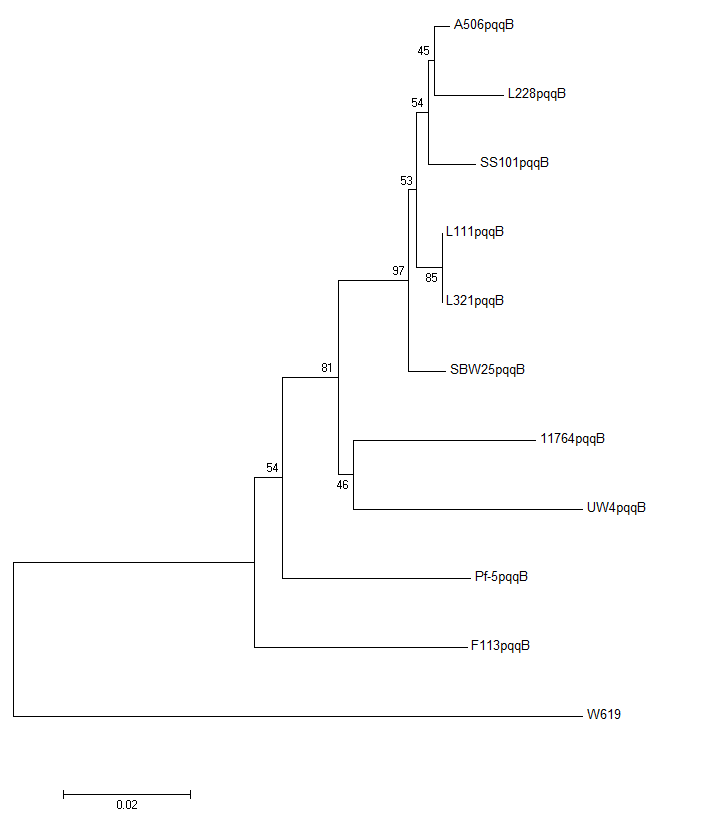


**Figure 1**: Neighbor joining phylogenetic tree predicting the relationships among the PqqB proteins in strains L228, L321 and L111 with those of published *Pseudomonas* genomes. (bootstrap values presented at the nodes).


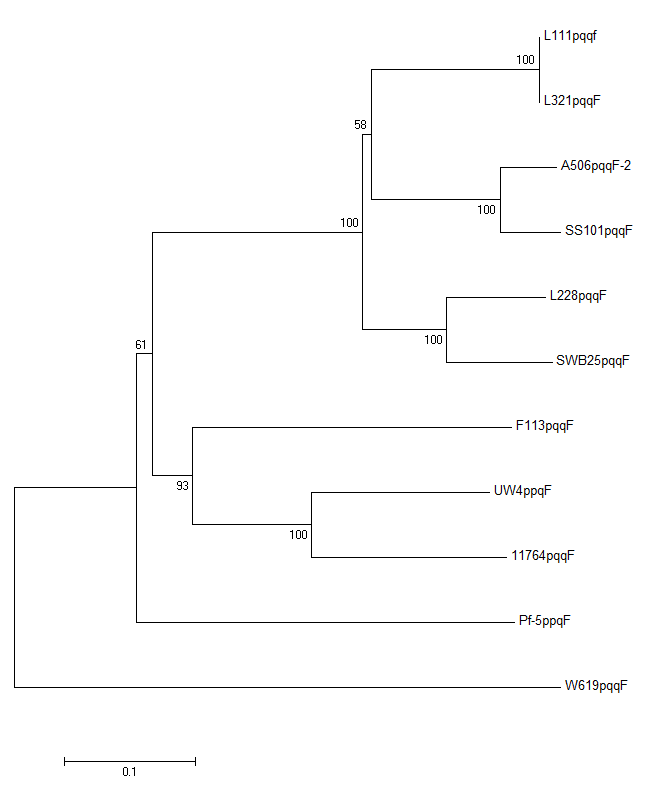


**Figure 2**: Neighbor joining phylogenetic tree predicting the relationships among the PqqF proteins in strains L228, L321 and L111 with those of published *Pseudomonas* genomes. (bootstrap values presented at the nodes).


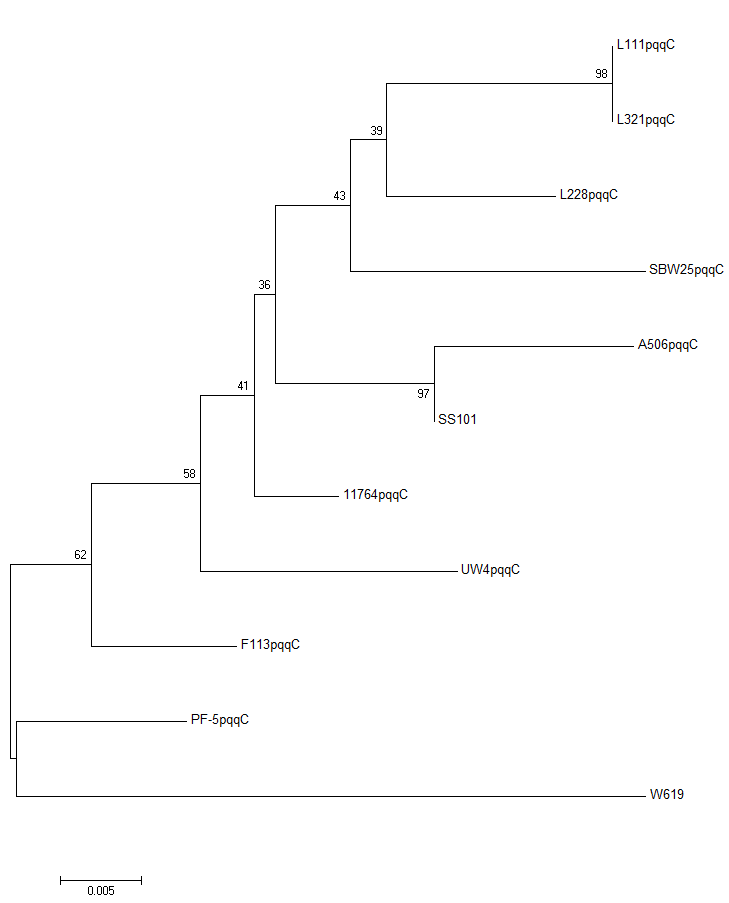


**Figure 3**: Neighbor joining phylogenetic tree predicting the relationships among the PqqC proteins in strains L228, L321 and L111 with those of published *Pseudomonas* genomes. (bootstrap values presented at the nodes).


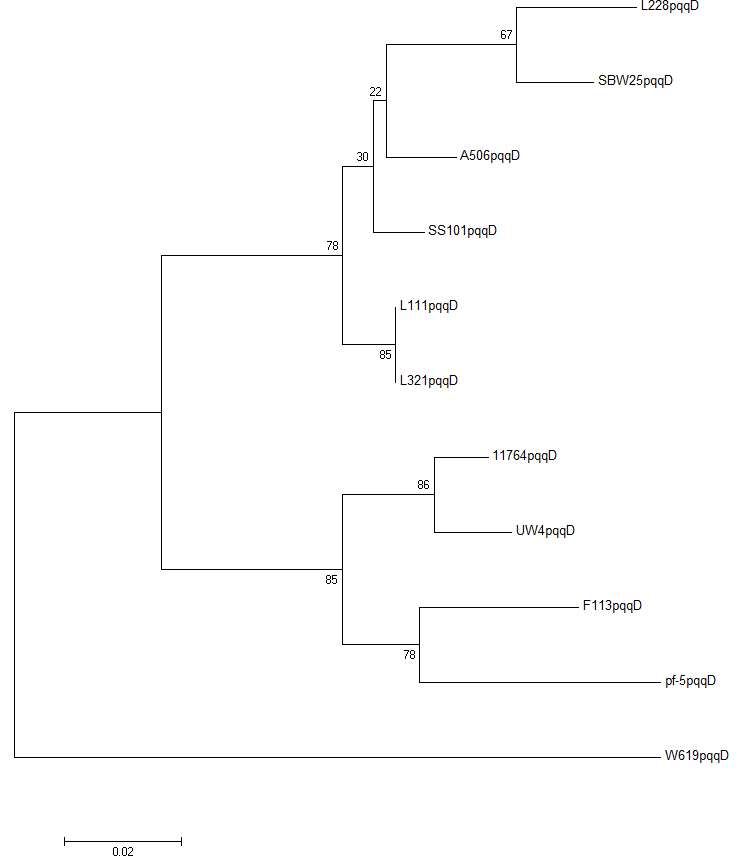


**Figure 4**: Neighbor joining phylogenetic tree predicting the relationships among the PqqD proteins in strains L228, L321 and L111 with those of published *Pseudomonas* genomes. (bootstrap values presented at the nodes).


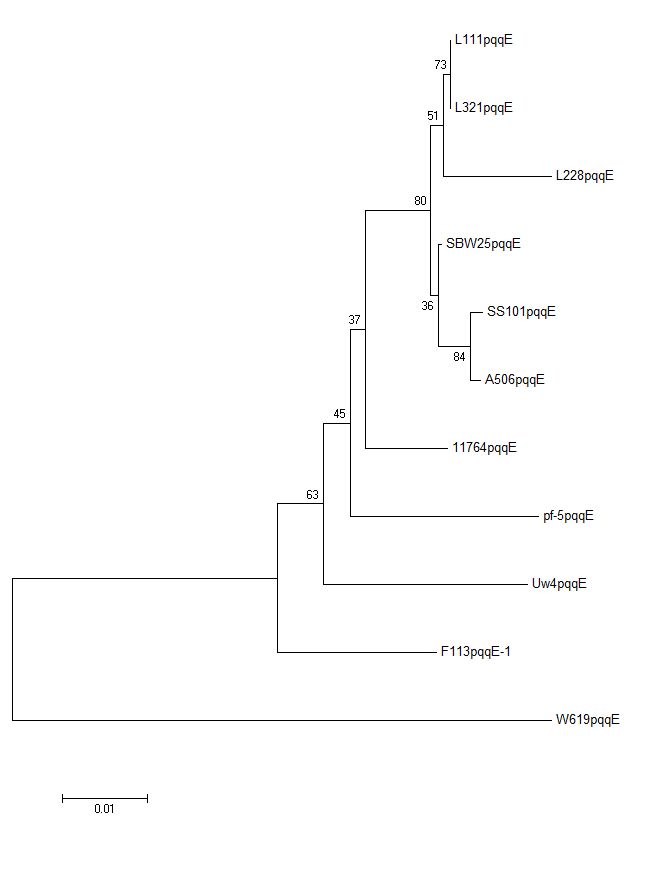


**Figure 5**: Neighbor joining phylogenetic tree predicting the relationships among the PqqE proteins in strains L228, L321 and L111 with those of published *Pseudomonas* genomes. (bootstrap values presented at the nodes).

**
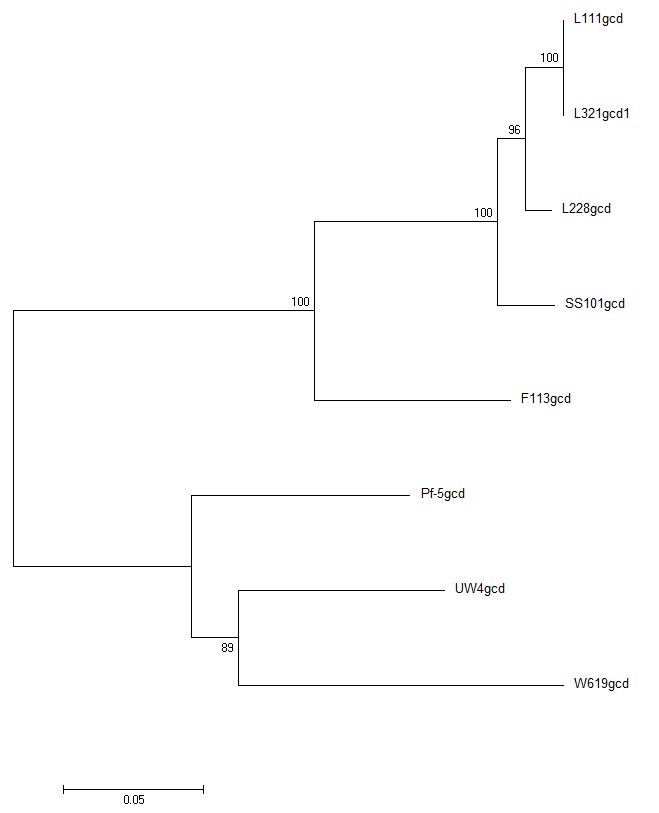
**

**Figure 6** Neighbor joining phylogenetic tree predicting the relationships among the *Gcd* proteins in strains L228, L321 and L111 with those of published *Pseudomonas* genomes. (bootstrap values presented at the nodes).

**
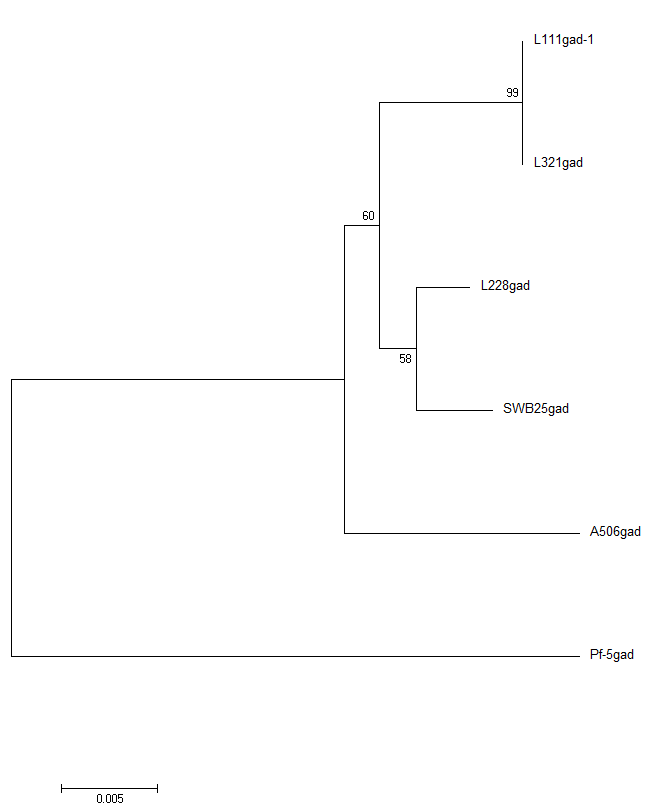
**

**Figure 7** Neighbor joining phylogenetic tree predicting the relationships among the *Gad* proteins in strains L228, L321 and L111 with those of published *Pseudomonas* genomes. (bootstrap values presented at the nodes).
